# Sustainable Synthesis of Multifunctionalized Amoxicillin-Loaded Biopolymer Foams

**DOI:** 10.1021/acsomega.5c00442

**Published:** 2025-04-11

**Authors:** Kerim Emre Öksüz, Saynur Arslan

**Affiliations:** † Department of Metallurgical and Materials Engineering, Sivas Cumhuriyet University, Sivas 58140, Türkiye; ‡ Institute of Science and Technology, Department of Bioengineering, Sivas Cumhuriyet University, Sivas 58140, Türkiye

## Abstract

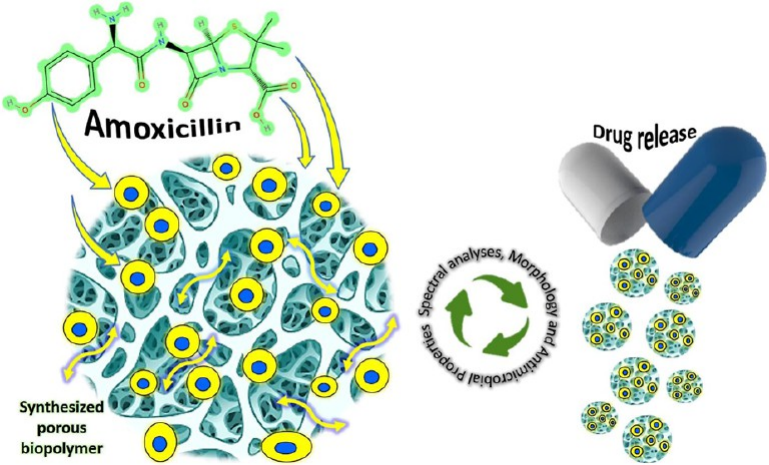

The development of biocompatible biopolymer foams loaded with antibiotics is crucial to advancing drug delivery systems in biomedical engineering. These materials offer controlled drug release and specialized functionalities for improved therapeutic outcomes. This study presents the development and characterization of antimicrobial polymeric biofoam materials loaded with the drug amoxicillin (AMX). The sustainable synthesis of these biopolymer foams involves a cost-effective, eco-friendly method that incorporates natural starch within poly(vinyl alcohol) (PVA) through an aldehyde cross-linking/stabilizing process. The highly porous structure of the biofoams enabled effective impregnation of the AMX drug using an innovative process involving ultrasonication and vacuum pressure to maximize efficiency and minimize biomaterial loss. The findings demonstrate the potential of these PVA/starch-based biofoams as versatile drug delivery systems with desirable physicochemical and biological characteristics. Detailed investigations were conducted to evaluate morphological features, chemical properties, swelling behavior, in vitro biodegradability, drug release profiles, cell culture, and antimicrobial activity tests of the prepared biofoam samples. Investigating the effect of controlled loading of AMX under laboratory conditions on its release profile and studying its biodegradation in various environments over time represent a critical aspect of this research. The optimal release profile under physiological conditions and the potent inhibition of bacterial growth against *Escherichia coli* and *Staphylococcus aureus* microorganisms by AMX-loaded biofoam materials highlight their potential for biomedical applications. These materials show promise for the in vivo administration and local treatment of bacterial infections.

## Introduction

Porous polymer structures have long been favored as functional materials in various technological fields, particularly in biomedical applications, due to their diverse functionalities. These versatile biomaterials find broad applications in filtration, purification, water desalination, hemodialysis membranes, tissue engineering scaffolds, and as base materials for biosensors, biomedicines, and catalysts. Commercially available porous biopolymers exhibit promising properties, including low surface tension, excellent wetting capacity, and other remarkable characteristics, making them particularly attractive for pharmaceutical and medical applications.^1,2^ The environmental impact of discarding petroleum-based foams has become a significant concern, paving the way for more sustainable and biodegradable alternatives. In this context, both synthetic and natural polymers derived from renewable resources, such as natural starch and poly(vinyl alcohol) (PVA), have emerged as promising options. These materials could effectively replace petroleum-based polymers in various applications.^3^

PVA is a well-recognized synthetic polymer that holds significant industrial importance due to its numerous favorable properties. It is nontoxic, compatible with biological systems, soluble in water, semi-crystalline, completely biodegradable, and comparatively less expensive than the currently predominant foam material, polyurethane (PU). Additionally, PVA showcases versatility through its range of average molar mass and degree of deacetylation, making it adaptable for a wide array of applications. Thus, ongoing research on PVA foams explores their combination with natural polymers like chitosan (CS), starch, and cellulose.^4−6^ Starch is particularly noted as a promising modifier for PVA to boost its biodegradation rate and curtail its cost, despite showing only partial compatibility with PVA. Studies, albeit limited, have started to explore physically cross-linked PVA/starch foams.^7^ The potential of a nanofibrous starch-based scaffold for wound healing, as reported by Waghmare and colleagues, is a noteworthy example in the literature.^8^ A biocompatible biomaterial must fulfill several conditions, including being non-toxic, non-carcinogenic, chemically inert, free from leachable pollutants, and mechanically stable, while displaying desirable surface qualities. Polymers have proven to be effective in medication delivery systems, a field receiving increasing attention in recent studies.^9^ The practice of blending starch with synthetic polymers, such as PVA and aliphatic polyesters, is commonly employed to achieve desired performance characteristics. Here, starch granules expedite the plastic matrix’s breakdown, a feature particularly useful for creating products such as hydrogels, bone cements, drug delivery systems (DDSs), and bone repositioning or fixation tools.^10^ The benefits of sponge antibacterial dressings, such as superior exudate absorption, efficient oxygen transmission, and excellent biocompatibility, make them ideal for wound healing and potential drug delivery systems. PVA/starch foams, due to their biocompatibility, biodegradability, and controlled drug absorption and release capabilities, have shown remarkable potential for drug delivery systems. Their porous architecture increases their surface area contact with biological fluids, facilitating improved drug absorption and release.^11^ Drug delivery and therapeutics in medical applications are constant subjects of scientific research, with the effectiveness of drug delivery systems (DDSs) being a major concern. Key challenges include side effects, anticancer effects, toxicity, lack of sensitivity, low bioavailability, short-term delivery, and rapid degradation.^12−16^ Innovative studies on DDSs based on biopolymers have been conducted due to their numerous advantages. Biopolymers, synthesized from natural or plant-based sources,^17,18^ are easily biodegradable, containing carbon, oxygen, and nitrogen atoms. They offer renewability, biocompatibility, affordability, lower carbon emissions, and environmental friendliness. Studies have proven that biopolymers are non-toxic, non-carcinogenic, non-thrombogenic, and easy to remove.^19^ Additionally, the presence of hydroxyl, amino, or carboxyl functional groups in natural biopolymers provides superior chemical reactivity and adaptability compared with synthetic biopolymers and hydrogels used in DDSs.

Khan et al.^20^ developed chitosan–alginic acid hydrogels for antibiotic encapsulation and release in combination therapy using a cost-effective method to load vancomycin, ciprofloxacin, and amoxicillin (AMX). Their analysis revealed that drug loading and pH variations significantly influence the hydrogels’ structure and release profiles. Polymeric biomaterials offer advantages over traditional pharmaceutical formulations, such as controlled and extended release, localized administration, stimulated release, enhanced mechanical strength, and improved biocompatibility. Wang et al.^21^ highlighted the disadvantages of using hydrophobic starch from wheat, rice, corn, and potato as biomaterials, noting issues like low water solubility and nonuniform texture. They suggested chemical, physical, and enzymatic modifications to improve starch-based DDSs, overcome these disadvantages, and produce effective DDSs. Taskin et al.^22^ also studied the modification of physical properties and found that surface nanotopography strongly affects cell adhesion, migration, and other functions by producing polycaprolactone (PCL)/poly(ethylene oxide) (PEO) polymers via the electrospinning method. They found that nanotopography features increased the proliferation of human umbilical vein endothelial cells (HUVECs) compared with smooth polymeric films. Their analysis showed that these features enhanced the formation of nascent adhesion complexes, even in scaffolds with a high PEO content. In recent studies, Zhu et al.^23^ developed a one-pot method to prepare mesoporous silica nanoparticles using α-linolenic acid (α-LA) as a template, which showed antibacterial activity against *Staphylococcus aureus* and antimycobacterial activity against *Mycobacterium tuberculosis*. The materials, encapsulating isoniazid and rifampicin, demonstrated the potential for combating drug-resistant infections. In another study they conducted, they reported^24^ the fabrication of CTAB/lipophilic oleic acid-modified grafted mesoporous silica nanocomposites as a nanocarrier for encapsulating itraconazole (ITZ). The produced nanocomposite exhibited efficient ITZ loading and sustained release, showing favorable inhibitory effects against *Aspergillus fumigatus* and *Candida albicans*.

In this experimental study, we loaded the amoxicillin (AMX) drug, the active ingredient of which is AMX, into polymeric biofoams based on PVA/starch materials, and a simple and eco-friendly method was applied to fabricate porous PVA biofoams by mixing different kinds of starches. The synthesized biofoam, produced through a modification process involving aldehyde cross-linking and the addition of sodium carbonate and hydrochloric acid as pore-forming agents into the PVA-starch matrix, exhibits enhanced physical and biological properties. Its surface morphologies have been evaluated using field emission scanning electron microscopy (FE-SEM), and its chemical analytical measurements have been assessed through Fourier transform infrared (FT-IR) spectroscopy. Additionally, its swelling ratios, in vitro biodegradability indices, drug release rates, cell viability, and antimicrobial activity tests have been thoroughly investigated.

## Materials and Methods

### Chemicals

Poly(vinyl alcohol) polymer (PVA, (C_2_H_4_O)_*x*_, 15% w/v; Mw = 60.000 g/mol), formaldehyde solution, 27–31% w/v (CH_2_O, with 10% (CH_3_OH)), hydrochloric acid (HCl) fuming 37%, and sodium carbonate (Na_2_CO_3_, 99.5% extra-pure) were purchased from Sigma-Aldrich (St. Louis, U.S.A.). Commercial corn, rice, and wheat starches were sourced from the local market in Türkiye. Amoxicillin anhydrous ((2*S*,5*R*,6*R*)-6-[[(2*R*)-2-amino-2-(4-hydroxyphenyl)acetyl]amino]-3,3-dimethyl-7-oxo-4-thia-1-azabicyclo[3.2.0]heptane-2-carboxylic acid (C_16_H_19_N_3_O_5_S; Mw = 365.4 g/mol; ≥98% purity)) was supplied from BioCrick BioTech (Chengdu, China). *Escherichia coli* (ATCC 25922) and *S. aureus* (ATCC 29213) type cultures were purchased from Sigma-Aldrich (St. Louis, U.S.A.). Both microorganisms were maintained as frozen stocks at −80 °C in 20% glycerol. Ultrapure water was prepared by using a Milli-Q50 SP reagent water system (Millipore Corporation, MA, U.S.A.) for the synthesis of all samples. All other reagents and supplies used for in vitro experiments were of analytical grade and were procured from Merck KGaA (Darmstadt, Germany), Thermo Fisher Scientific (Massachusetts, U.S.A.), and Bayer AG (Leverkusen, Germany).

### Sustainable Synthesis of Polymeric Biofoam Samples

The sustainable synthesis of the PVA/starch biofoam was accomplished by adapting and applying the procedure outlined in our previous research.^2^ Briefly, 10 g of PVA was dissolved in 90 g of distilled water (dH_2_O) while stirring at 500 rpm and heating to 80 °C for 1 h to produce a 10 wt % PVA solution. The prepared PVA solution was cooled to below 65 °C. Then, a starch blend (comprising approximately 1.65 g each of rice, wheat, and corn starch, for a total of 5 g) was progressively added to the PVA solution. This mixture was stirred for 1 h at 65 °C until a homogeneous aqueous solution was obtained. Following this procedure, the mixture was agitated for 1 h at 65 °C until a uniform solution was formed. Subsequently, 1.5 g of Na_2_CO_3_ was added as a foaming agent, which resulted in an observable increase in the solution’s viscosity. Then, 30 mL of a formaldehyde solution (CH_2_O, 27–31% w/v with 10% CH_3_OH) was incorporated into the PVA/starch/Na_2_CO_3_ mixture to aid in stabilizing the foam structure, and mechanical stirring continued at 500 rpm for an additional 5 min. The stirring speed was then increased to 2500 rpm for a final 5 min. After CH_2_O was added to the PVA–starch–Na_2_CO_3_ solution, mechanical stirring was maintained for 5 min to promote the reaction and prevent volatilization of the components. Finally, 25 mL of fuming HCl (37%) was added to induce the immediate formation of froth. The reaction was allowed to proceed for 20 s, after which the stirring was stopped. During the forming process, vessels of adequate size were used, considering that the foaming can expand up to four times the volume of the original suspension. All experimental work during the synthesis was conducted under a fume hood. Once the mixture was thoroughly blended, it was transferred into molds and left to dry at 55 °C overnight. The dried product underwent a thorough rinsing with dH_2_O until the rinse water was clear (dH_2_O, pH ∼ 7 at 25 °C), indicating that most of the residual HCl, CH_2_O, and starch had been eliminated. The final stage consisted of drying the cleaned polymer/starch mixture once more at 60 °C for a duration of 4 h, and then they were kept in a desiccator before being tested. Potential formation of hydrogen bonds between starch and PVA is given in Figure S1 (Supporting Information), and the diagrammatic representation details of the synthesis process for the PVA/starch biofoam are provided in Figure S2 (Supporting Information).

### Drug (AMX) Loading to Biofoam Samples

The highly porous structure of the synthesized polymeric biofoams enables easy penetration and absorption of an AMX solution. Biofoam samples, each weighing 2 g, were immersed in a dimethyl sulfoxide (DMSO) solution with AMX (100 mg/mL; 273.5 mM) prepared in a closed sterile flask. The biofoam samples with AMX solution were subjected to 45 min of ultrasonication at an ultrasound frequency of 25 kHz in an ultrasonic bath maintained at a temperature of 30 to 35 °C. During the impregnation of AMX into biofoams, a flask which contained samples and an AMX solution was placed on the setup, and a vacuum pump was used to create a constant vacuum pressure of 9 × 10^–1^ bar to enhance impregnation. The saturated biofoam samples were then extracted from the AMX solution and left to stabilize at room temperature in a sterile cabinet.^25^

### Exploring the Structural Features of Biofoam Samples

The microstructure and surface characteristics of the synthesized biofoam surfaces and cross sections were explored in detail using FE-SEM (Tescan Mira3 XMU, Brno, Czechia). All samples were affixed to aluminum stubs and coated with a thin Au/Pd film for 1.5 min within an Ar gas environment using a Quorum Q150R ES sputter coater (Birmingham, UK). Composition analysis of biofoams was performed using energy-dispersive spectroscopy (EDXS) apparatus attached to a scanning electron microscope (INCA IE 350, and U.K.).^26^

### Fourier Transform Infrared (FT-IR) Spectroscopy

Before conducting FT-IR spectroscopy, the biofoam samples were initially air-dried. The spectra were collected using a Bruker Alpha II FT-IR spectrophotometer (Germany) with 128 scans acquired in the wavenumber range from 400 to 4000 cm^–1^. By comparing the spectra of the biofoam samples, variations in their chemical composition and bonding were identified, offering valuable insights into their physical properties and potential applications.^27^

### Swelling Behaviors of the Biofoam Samples

The biofoams were measured and immersed in a phosphate-buffered saline (PBS) solution with a pH of 7.4 at ambient temperature for various durations. The weight of the biofoam samples was assessed at regular intervals. Subsequently, the biofoams were transferred to PBS and statically cultured at 37 °C for 72 h. Excess surface H_2_O was absorbed using thin filter paper, and this process was repeated until the sample reached saturation. At hours 1, 2, 4, 8, 24, 48, and 72, the swelling ratio and sol fraction of the biofoam samples were determined using eq 1
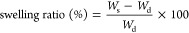
1Here, *W*_s_ and *W*_d_ represent the weight of samples after swelling in PBS and the weight of dried samples after swelling, respectively.^28^ The swelling ratio is a critical parameter that quantifies the fractional increase in the weight of the biofoam due to water absorption. As the biofoam samples undergo swelling and eventual degradation, the swelling ratio decreases over time, signifying the loss of polymer and reflecting the extent of sample degradation. Serving as a fundamental indicator of biofoam behavior, the swelling ratio plays a central role in assessing the biomaterials water-absorption capacity and structural stability.

### In Vitro Biodegradation Assessment

For the assessment, dried biofoam samples (30 °C for 24 h) were prepared and weighed (0.01 g). These samples were then immersed in 3 mL of PBS (0.1 M, pH 7.4, maintained at 37 °C) at regular intervals over a 21 day experimental period. Once removed at specified time intervals, the samples were gently blotted with a soft paper towel to eliminate surface water and subsequently dried in an oven before being weighed again by using a calibrated scale. The resultant weight was duly noted for record. To affirm the reliability and reproducibility of the results, all experimental procedures were meticulously carried out in duplicate. The rate of degradation was determined through the application of eq 2.^29^
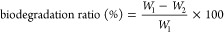
2where *W*_1_ and *W*_2_ represent the weight of biofoams prior to and following in vitro biodegradation, respectively.

### Cell Culture Test

L929 mouse fibroblast cells (ATCC, CCL-1) were used in the study. The cells were incubated in media supplemented with 10% fetal bovine serum and 100 U/mL penicillin–streptomycin at 37 °C with 5% CO_2_. The culture medium was replaced every 2–3 days. Prior to testing, both synthesized samples were completely sterilized. Cell viability was determined using the MTT (3-(4,5-dimethyl-2-thiazolyl)-2,5-diphenyl-2*H*-tetrazolium bromide) assay. Cells (5 × 10^3^ cells/well) were seeded in 96-well plates with growth media, then treated with the prepared samples, and incubated for 24 h under standard cell culture conditions. After incubation, cells were exposed to 10 μL of MTT reagent in serum-free medium for 4 h, and the resulting formazan crystals were dissolved with dimethyl sulfoxide. Absorbance was measured at 520 nm for each well using a plate reader (Thermo Scientific, Multiskan FC). Cell viability was calculated as a percentage of the control test, which was considered to be 100%.^30^

### Antimicrobial Activity of Biofoam Samples

The antimicrobial potential of the biofoam samples was evaluated using the agar diffusion method. Indicator bacteria *E. coli* and *S. aureus* were cultivated twice in Mueller–Hinton broth prior to assessment. For the in vitro experiment, the concentration of the bacteria in the overnight cultures was adjusted to match McFarland Standard 0.5. Afterward, 1 mL of this adjusted bacterial culture was spread on preprepared Mueller–Hinton agar plates. After a waiting period of approximately 20 min for the agar surface to fully absorb the bacterial culture, the biofoam samples were positioned on the plates. The plates were then incubated for a duration of 24 ± 2 h at a temperature of 37 °C. Following the incubation period, the zones of inhibition around the biofoam samples were gauged, and the antimicrobial activity was computed by deducting the sample size from the diameter of the inhibition zone. For comparison purposes, a disk impregnated with 30 μg of AMX was used as a positive control (+) in the test, while a sterile blank disk was used as the negative control (−). Each assay was conducted in triplicate, and the reported results represent the average of these three trials.^31^

### Drug Release Profile Study

The objective of this research is to examine the in vitro drug release patterns exhibited by biofoam samples. Our primary focus is to gain insights into the kinetics and mechanisms governing drug release from the biofoam matrices under simulated physiological conditions. Through a thorough investigation of in vitro drug release, we aim to assess the viability of these biomaterial formulations as drug delivery systems for a wide range of therapeutic applications. Initially, biofoams were loaded with AMX at five different drug concentrations (2, 4, 6, 8, and 10 mg/mL), and the absorbance curves were calculated at a wavelength of 290 nm. Among the PVA/starch biofoams loaded with the drug at these concentrations, the one with the most optimal loading efficiency was selected. Subsequently, a drug release study was conducted on this biofoam at the selected concentration. To examine the drug release characteristics of AMX from the PVA/starch/AMX sample, PBS (pH 7.4) was used in a thermal shaker (Laboratory Shaking Water Bath, Being Lab, PLT Scientific Sdn Bhd, Malaysia). Biofoam samples, each weighing 10 mg, were placed into Eppendorf tubes containing 5 mL of PBS (pH 7.4) for the release step. Absorbance values were then recorded using a UV spectrophotometer (Thermo Scientific, Multiskan FC, UV–visible, U.S.A.), with the absorption wavelength for prepared samples set at 290 nm. The drug release studies were conducted until a stable state was reached with no further observable changes in the AMX concentration.^32^ The subsequent formula [eq 3] was used to compute the percentage of AMX released
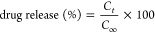
3where *C*_*t*_ and *C*_∞_ represent the concentration of the drug at time *t* and its complete release, respectively.^33^

### Statistical Analyses

The collected data were presented as means ± standard deviations (SDs) based on at least three (*n* = 3) independent measurements. All data were statistically analyzed using one-way ANOVA followed by the Bonferroni *t*-test. Statistical significance was determined at *p ≤ 0.05 by using Origin Pro 9.0 software program.

## Results and Discussion

### Fourier-Transform Infrared Spectroscopy

**Figure 1:**
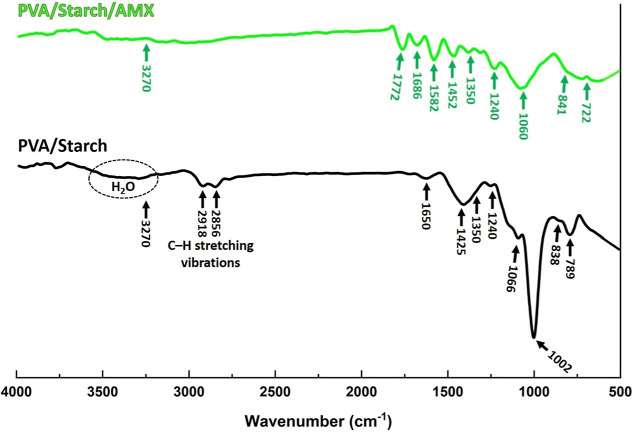
FT-IR spectra of the PVA/starch and PVA/starch/AMX surface compositions. These spectra showcased distinct peaks in the biofoam samples, providing detailed data regarding the presence of AMX drug components. Within the FT-IR spectra, one could readily identify characteristic peaks associated with both starch and AMX, confirming their effective integration into the biofoam matrices.

The FT-IR spectra for the PVA/starch and PVA/starch/AMX biofoams are displayed in Figure 1. Starch and PVA can absorb moisture from the environment and retain it due to the presence of –OH groups within their polymer chains.^34^ Through their hydroxyl groups (–OH), PVA and starch can interact strongly. The process of combining PVA with different kinds of starch enhances the water absorption capacity of the biofoam samples. This is primarily because of the inherent hydrophilic properties of starch, which is inherently polar due to the presence of –OH groups. These groups enable the formation of hydrogen bonds with water, influencing the water’s resistivity within the mixtures.^35^ The PVA/starch biofoams FT-IR spectra show a distinctive –OH stretch band at 3270 cm^–1^.^36^ The transmittance observed at 1240 cm^–1^ could be associated with specific vibrational modes in the molecule, possibly related to C–H functional groups. These groups are prevalent in substances like PVA and certain forms of starch.^37^ The bands in the region of 1066 cm^–1^ correspond to C–O vibrations of C–OH groups, while the bands around the regions of 2918 and 2856 cm^–1^ are related to C–H stretching vibrations. The starch spectra exhibit a distinctive broad band O–H deformation bending at 1650 cm^–1^. A transmittance of 1002 cm^–1^ characterizes the C–O stretching vibrations present in glucose rings. The significant decrease in the absorption band at 1002 cm^–1^ in the FT-IR spectrum of the AMX-loaded biofoam suggests an interaction between AMX and the polysaccharide matrix. This peak is attributed to C–O stretching vibrations in starch, and its reduction in intensity indicates that AMX may be interacting with the hydroxyl and ether groups in the starch structure through hydrogen bonding. This interaction could alter the vibrational modes of the polymer network, leading to the observed decrease in the peak intensity. CH–CH_2_ vibrations are indicated by the bands at 1425 and 1350 cm^–1^, while the –CH_2_ bending is shown at 838 cm^–1^ in the FT-IR spectra.^38^ A very subtle absorption band, connected to the vibrational modes of starch polysaccharides and disaccharides, is seen in the 789 cm^–1^ range of the FT-IR spectra. In the FT-IR of AMX in isolation, characteristic band peaks were observed at 1772 cm^–1^, corresponding to the C=O stretching in the β-lactam ring, at 1686 cm^–1^ associated with the C=O stretching in the amide, and at 1582 cm^–1^ for the asymmetric stretching of the carboxylate group (RCO^–2^).^39^ The existence of functional groups in AMX was affirmed by the respective peaks observed at 1582, 1686, and 1772 cm^–1^ in the FT-IR spectra.^40^

### Microstructural and Morphological Characterization (FE-SEM)

**Figure 2:**
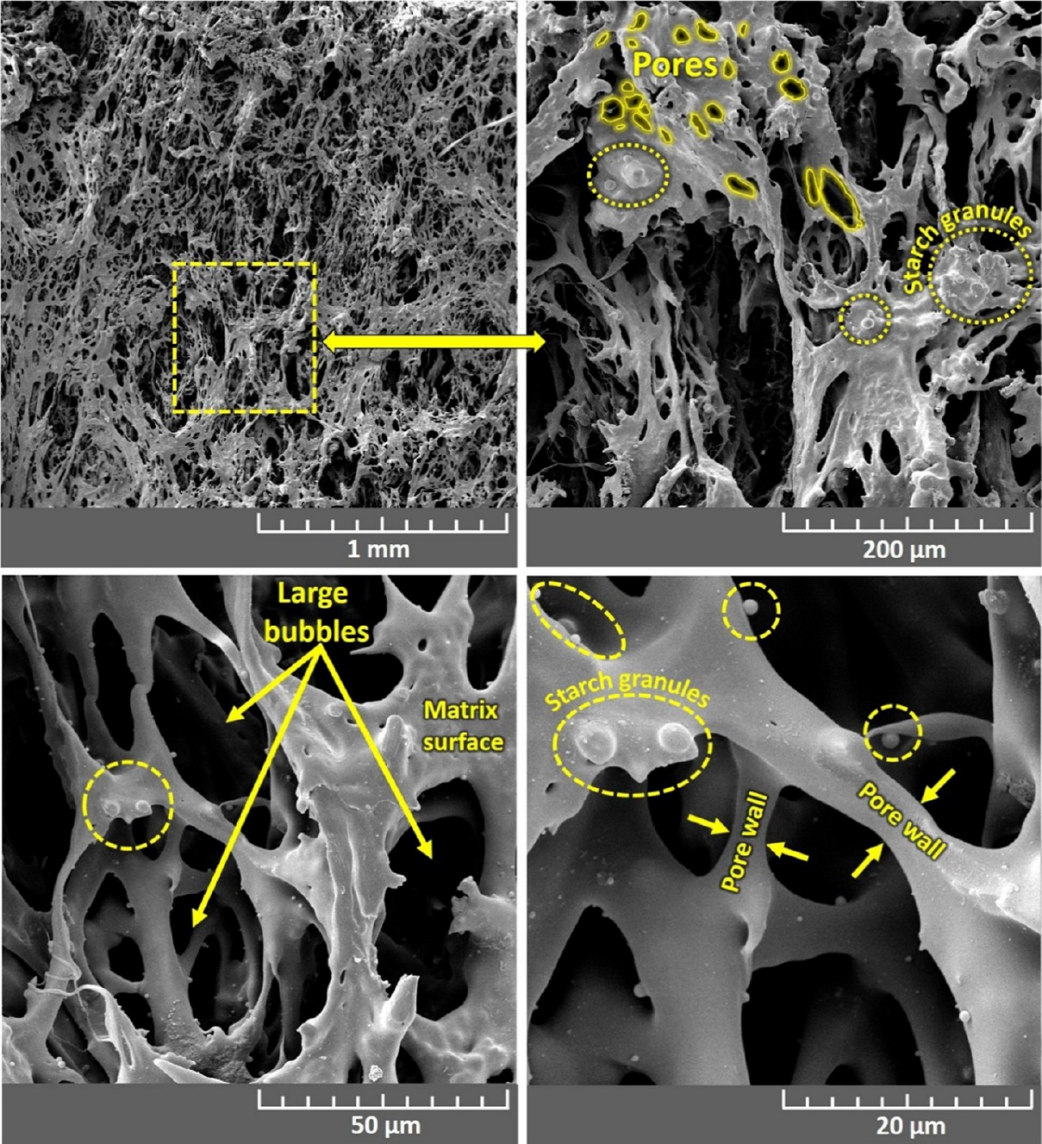
FE-SEM micrographs of cross-section surface morphology of PVA/starch biofoams. High-resolution FE-SEM micrographs clearly capture the intricate surface morphologies of PVA/starch biofoams.

**Figure 3:**
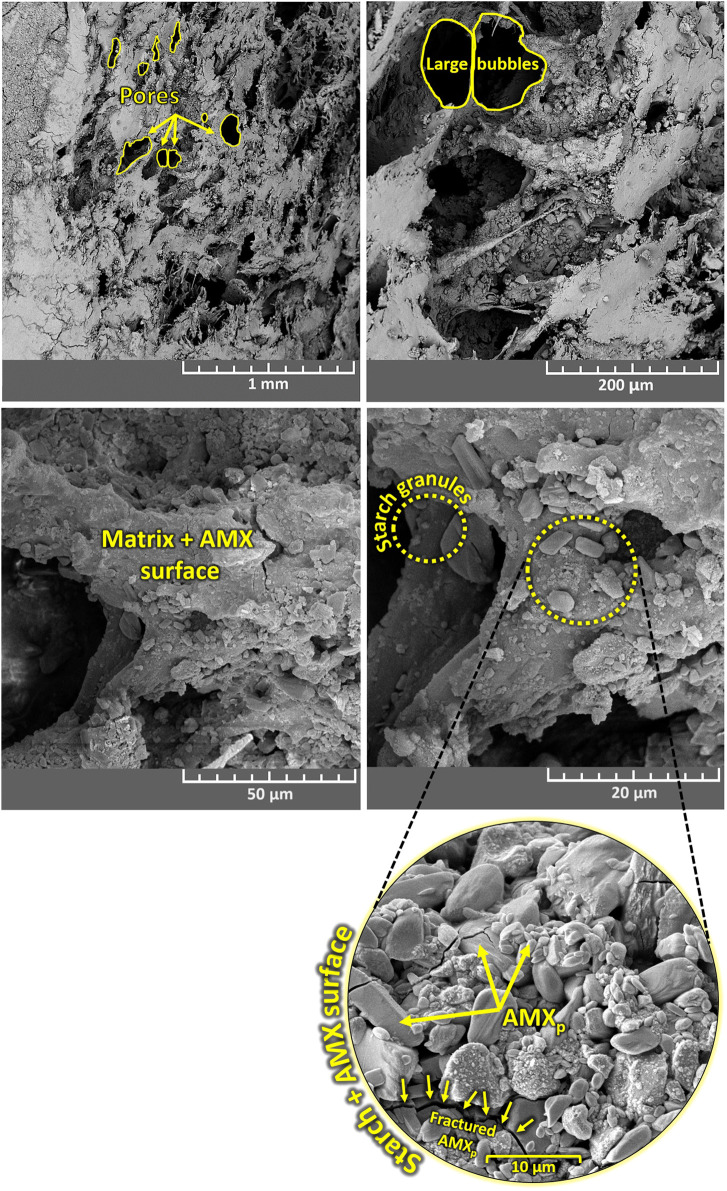
FE-SEM micrographs of cross-section surface morphology of PVA/starch/AMX biofoams. High-resolution FE-SEM micrographs offer a detailed examination of the cross-sectional surface morphology of PVA/starch/AMX biofoams. These micrographs strongly showcase the effective integration of AMX particles into the PVA/starch matrix. Notably, the appearance of AMX drug crystals within the porous foam matrix signifies the uniform dispersion of the drug throughout the foam. Further insights into the high-magnification morphology of the AMX drug are presented in figure as an inset, revealing a distinct rectangular and irregular shape. This comprehensive analysis provides a clear and insightful understanding of the structural characteristics and distribution of the AMX drug within the PVA/starch/AMX biofoams.

The morphology, microstructure, and pore size of PVA/starch and PVA/starch/AMX biofoams were analyzed by FE-SEM. Figure 2 shows FE-SEM micrographs of the PVA/starch biofoam surfaces, while Figure 3 displays FE-SEM micrographs of the PVA/starch/AMX biofoam surfaces. Additionally, inset FE-SEM micrographs in Figure 3 depict the detailed interface region in the cross-section of the PVA/starch/AMX biofoam surfaces. Upon examination of the morphological features presented in Figure 2 for the synthesized biofoams, a distinct fibril structure pervades the entire matrix. A high-magnification investigation of this fibril structure reveals the presence of spindle-like-shaped pores and partially spherical pores. Notably, lump-shaped starch granules are interspersed throughout the biofoam matrix, manifesting as particles. An integral contributor to the formation of discernible pores within the biofoam structure is Na_2_CO_3_, which was introduced as a foaming agent during the synthesis process. The active role of Na_2_CO_3_ is evidenced in the pronounced porosity observed, highlighting its influence in shaping the overall architecture of the biofoam. This strategic incorporation of Na_2_CO_3_ not only enhances the structural characteristics but also underscores its pivotal role in the successful fabrication of the biofoam with distinctive morphological features.^41^

The clarity of Figure 3 unequivocally explains the substantial impact of drug-polymer contamination on the morphology and size of the biofoams. Taking the example of the PVA/starch/AMX biofoam, it manifests a noticeably rougher structure compared to its PVA/starch counterpart, a characteristic likely attributed to the accumulation of AMX particles on its surface. Examining the structure of the PVA/starch/AMX biofoam, as illustrated in Figure 3, reveals a distinct open-cell configuration. This structural feature suggests the foam’s potential efficacy in efficiently entrapping the drug. Further scrutiny through high-magnification inset FE-SEM photographs in Figure 3 clearly demonstrates the homogeneous distribution of drug particles within the biofoam matrix. The observed roughness in the PVA/starch/AMX biofoam and its unique open-cell configuration present valuable insights into the potential applications of these biomaterials, particularly in drug delivery systems where effective entrapment and controlled release are paramount.

PVA/starch and PVA/starch/AMX biofoams exhibit slight distinctions in the trends of porosity size reduction when compared to the results obtained from the FE-SEM analysis. This difference in behavior is likely attributable to the presence of incorporated AMX particles, which effectively coat the surfaces of the biofoam pores. The pore sizes were determined to be 149 ± 36 μm and 196 ± 28 μm for PVA/starch/AMX and PVA/starch biofoam samples, respectively. This measurement involved the analysis of at least 100 pore diameters sourced from two distinct images of the same magnification, employing the linear intercept method.^42^ The obtained data can explain the impact of AMX incorporation on the porosity characteristics of the biofoams, revealing an influence on their structural attributes compared to the PVA/starch samples. Due to its homogeneous microstructure, the synthesized biofoam could effectively load AMX at concentrations of 2, 4, 6, 8, and 10 mg/mL. The high pore surface area enabled loading of a high amount of the AMX drug. There were no significant structural changes observed in the biofoam before and after loading with AMX, as presented in Figures 2 and 3.

### EDXS Analyses

The composition of the compounds/particles present on the surface of the PVA/starch and PVA/starch/AMX biofoam samples was analyzed using FE-SEM–EDXS spectra. These spectra, along with their corresponding calculated values, are presented in Figure S3 (Supporting Information). As depicted in Figure S3, the EDXS spectra for both samples indicated a rich composition of carbon and oxygen, elements commonly found in starch and polymer compositions. Notably, the EDXS results for PVA/starch/AMX showed inclusion peaks of sodium, nitrogen, sulfur, and chlorine, attributed to the typical composition of AMX. The presence of S and Na verified the incorporation of AMX into the foam. S contributes to the composition of the thiazolidine ring (6-aminopenicillanic acid), a distinctive feature of penicillin derivatives,^43^ while Na is associated with the salt form of AMX. This observation supports the differences in structure seen within the FE-SEM and FT-IR results when compared with PVA/starch samples.

### Swelling Behavior Tests

**Figure 4:**
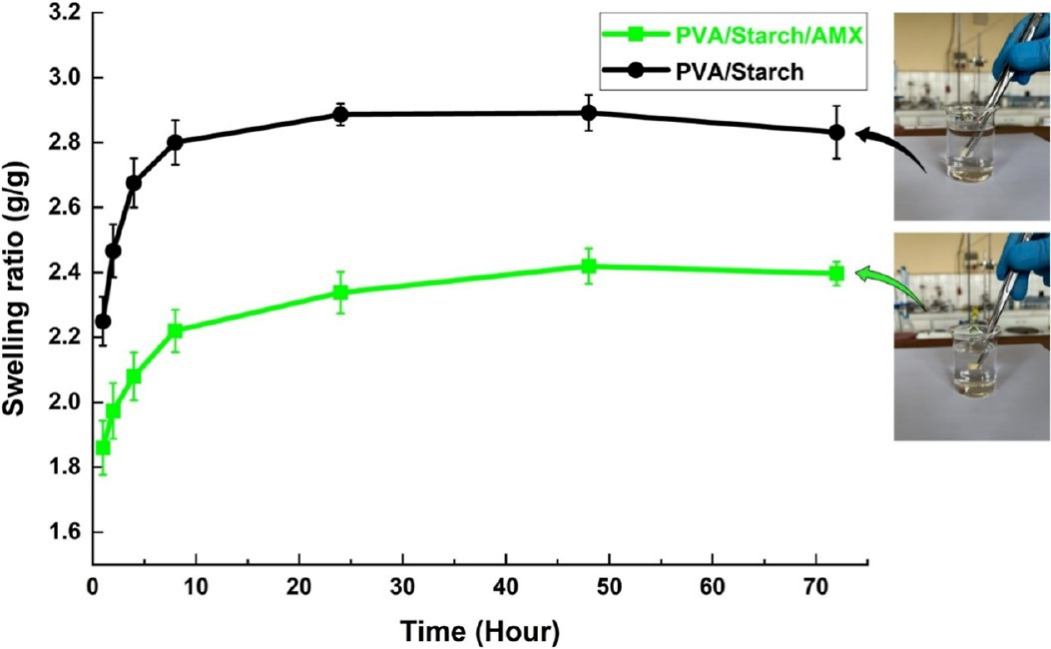
Swelling behaviors as a function of time for biofoam samples in PBS at pH 7.4, each characterized by distinct compositions. The error bars in the graph denote the standard deviation, and the experiment was conducted in triplicate (*n* = 3).

The hydrophilic functional groups of PVA and starch contributed to the swelling observed in the foam samples. Swelling is influenced by the ionization pattern in PBS and the hydrogen bonding interactions among H_2_O molecules. The hydrophilic groups of PVA and starch contributed to swelling of the foam samples. The extent of swelling was influenced by the ionization pattern in PBS and the hydrogen bonding interactions between water molecules. The swelling curves of synthesized foams of different formulations are presented in Figure 4. As the immersion time increased from 1 to 24 h, the PVA/starch foams swelled faster, and the equilibrium swelling ratio was enhanced accordingly compared to the PVA/starch/AMX. This occurred as a result of the separation of intertwined polymeric chains and disruption of hydrogen bonding between polymer molecules. With longer immersion times and in polymer foams without loaded AMX, the increased chain mobility facilitated expansion of the network. The hydrophilic functional groups present in both PVA and starch played a pivotal role in the swelling process of the foam samples. The extent of swelling was intricately influenced by the ionization patterns within PBS and the hydrogen bonding dynamics among water molecules. As the immersion time progressed from 1 to 24 h, a notable acceleration in the swelling rate of PVA/starch foams occurred, resulting in an augmented equilibrium swelling ratio compared to the PVA/starch/AMX foam sample. This phenomenon was associated with the separation of interpenetrated polymeric chains and the disturbance of hydrogen bonding among the polymer molecules. With a prolonged immersion time and in the absence of AMX in the polymer foams, an increase in chain mobility was observed, thereby facilitating the expansion of the network structure. This increased chain mobility underscored the pivotal role played by the immersion duration and the absence of AMX in influencing the overall swelling characteristics of the foam. The extent of swelling can be influenced by factors such as pH, temperature, chemical composition, and the amount of cross-linking agent.^44^ The enlarged biofoams maintained their structural integrity throughout the swelling process, showing no signs of visible breaking or fracturing. These biofoams were still manageable even when they reached their maximal swelling capacity, highlighting their potential use in biomedical applications. The elevated porosity of starch-based sponges facilitated a substantial degree of swelling by promoting a greater interaction between the highly hydrophilic chains of the foams and the surrounding medium. During the initial stages of experimental evaluation, a consistent increase was observed in the swelling capacity of the PVA/starch foam. This observed phenomenon could potentially be attributed to the time required for the sponges to reach equilibrium.

The biomaterial foam, comprised of PVA/starch, demonstrated an elevated degree of swelling, possibly linked to the inclusion of corn/rice/wheat starch. In the absence of the AMX drug, the foams experienced a roughly 4-fold weight increase during the swelling studies at pH 7.4. A gradual rise in swelling was noted within the initial 8 h, stabilizing after 48 h. As previously discussed, various factors impact the swelling behavior of foam samples, including the hydrophilic characteristics of the polymer structure, the concentration of cross-linking agents, and the flexibility of the polymer structure. The addition of starch enhances the hydrophilicity of the polymer system, leading to an improved swelling capability. This enhanced swelling capacity is particularly beneficial in drug delivery applications, where the polymer’s swelling behavior can control the release of the drug. Therefore, there exists a direct relationship between swelling capacity and the amount of drug released upon reaching equilibrium.^45,46^

In previous studies, amoxicillin-loaded films composed of poly(vinyl alcohol)-*g*-poly(acrylamide) with varying compositions were synthesized by using the graft copolymerization method. The swelling-controlled drug release behavior of the amoxicillin-loaded hydrogel was examined under in vitro conditions, and the effects of various factors, including the chemical composition of the grafted hydrogel, the % of drug loading (amoxicillin), and the pH and temperature of the release medium, were analyzed in relation to the drug release profiles. They observed that the swelling ratios of the prepared hydrogels increased with rising ambient temperature from the first hour until the end of the fourth hour. Additionally, they found that the swelling ratio of the antibiotic-loaded hydrogels was approximately 2.75 g/g after 1 h at 35 °C.^47^ Tran Vo et al.^48^ synthesized and characterized hybrid hydrogels to precisely regulate drug release. Gamma irradiation was employed as an environmentally friendly approach to produce hydrogels from high-molecular-weight chitosan (CS) and PVA without the use of toxic chemicals. An increase in PVA concentration and radiation dose up to 25 kGy led to a significant enhancement in the mechanical strength and swelling capacity of the blended hydrogels compared with neat PVA and CS hydrogels. In deionized water, the swelling ratios of hydrogels with varying CS/PVA ratios (0/100, 25/75, and 50/50) were determined to be 1.4, 1.98, and 3.0 g/g, respectively, after 60 min of soaking. After 120 min, the swelling ratios increased to 1.5, 2.2, and 3.2 g/g, respectively. In another study, the swelling properties of hydrogel samples prepared by dissolving carrageenan (C), a family of sulfated polysaccharides, and PVA in a 1% NaOH, 0.5 M solution were examined. The swelling behavior of the hydrogels was characterized by considering factors such as the weight ratio, plasticizer type, heating time, and processing temperature. The percentage swelling ratio was determined by using the weight difference method. The results indicated that the optimal C/PVA weight ratio for maximizing the swelling ratio was 1.1 g/g.^49^ Additionally, the study reported that the swelling ratio of hydrogels incorporating polyglycerol as a plasticizer was significantly higher than that of hydrogels using glycerol.

### Hydrolytic/Enzymatic Biodegradation Tests

Following implantation, biomaterials introduced into the in vivo environment engage with the surrounding fluids through the initial absorption of water, thereby triggering the initiation of their biodegradation process. This absorption of water not only renders the material more flexible but also induces dimensional alterations.^50^ Conversely, higher water absorption typically expedites the hydrolysis process.^51^ The process of hydrolytic degradation manifests as an autocatalytic phenomenon, wherein the carboxylic (–COOH) groups derived from the hydroxyl acids generated play a pivotal role in catalyzing subsequent hydrolysis reactions. This self-perpetuating mechanism highlights the catalytic nature of the carboxylic groups as they not only initiate but also actively accelerate the ongoing hydrolysis process. This interplay of chemical reactions exemplifies the autocatalytic nature of hydrolytic degradation, highlighting the significant role played by –COOH groups in facilitating and amplifying the overall degradation process.^52^

**Figure 5:**
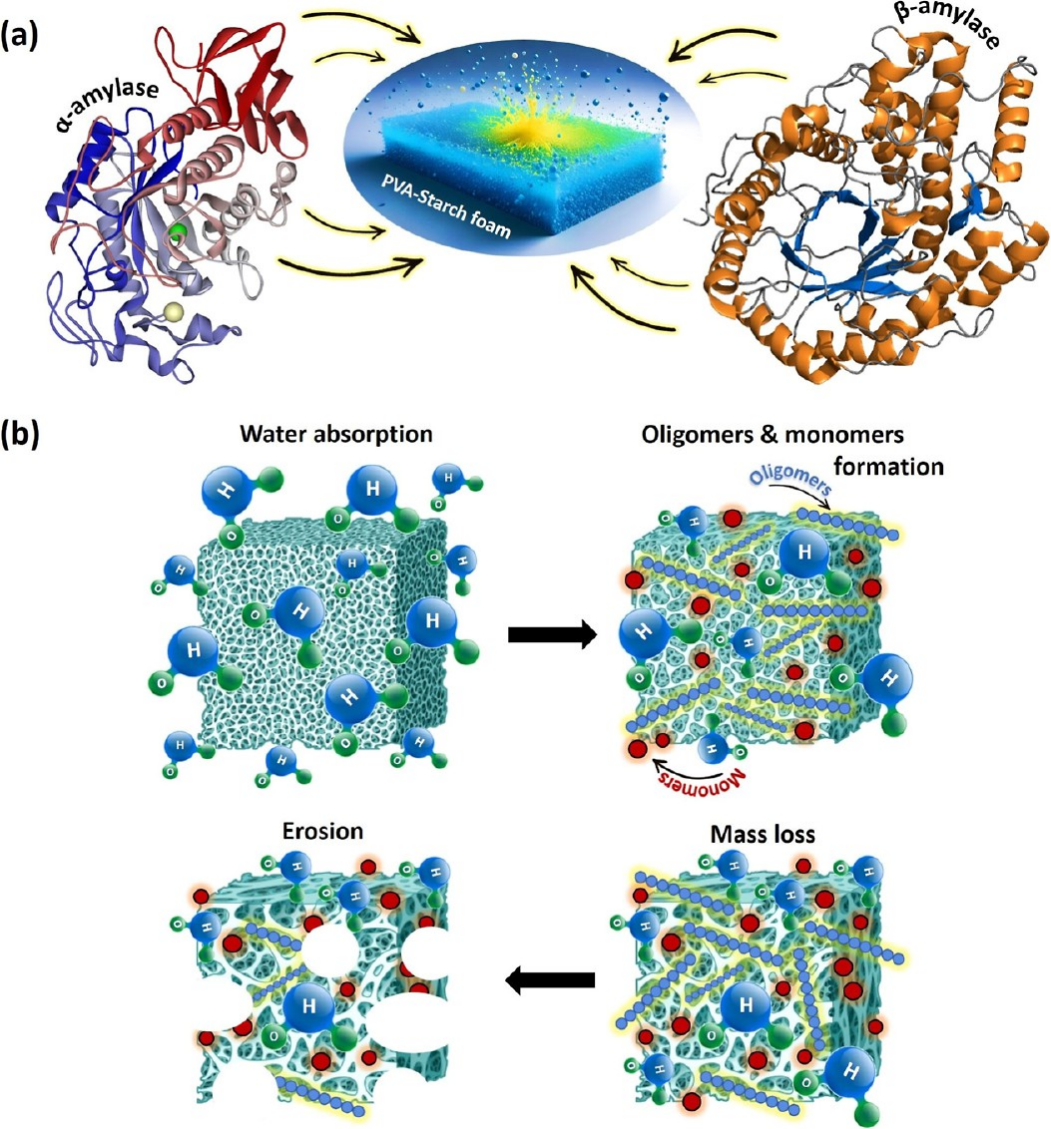
(a) The schematic mechanism of various enzymes involved in PVA/starch foam degradation. α-Amylase (RCSB PDB accession code 1SMD; the regions responsible for Ca-binding are identified), also known by its systematic name 4-α-d-glucan glucanohydrolase, catalyzes the hydrolysis of α bonds within large, α-linked polysaccharides like starch and glycogen. This process results in the production of shorter chains, namely, dextrins and maltose. β-Amylase (RCSB PDB accession code PDB 2xfr), also referred to as 1,4-α-d-glucan maltohydrolase, glycogenase, or saccharogen amylase, acts on the nonreducing end of substrates. β-Amylase facilitates the hydrolysis of the second α-1,4 glycosidic bond, resulting in the release of two glucose units at a time. starch is broken down into maltose by β-amylase, and both are classified under the glycoside hydrolase family.^62,63^ (b) The biodegradation process unfolds through a series of four sequential steps: (i) hydration involves the infiltration of an aqueous medium into the polymer matrix, disrupting secondary forces. (ii) Initial degradation occurs in the hydrated region, where covalent bonds in the polymer backbone undergo cleavage after hydrolysis, leading to a reduction in the polymer’s molecular weight. (iii) Further degradation continues as the molecular weight of the polymer diminishes to a threshold where the structural integrity can no longer be maintained. (iv) Solubilization or erosion marks the final stage, during which the polymer sheds weight, and fragments are further cleaved into soluble molecular entities within the medium.^64^

PVA stands out as an exceptionally versatile water-soluble polymer, uniquely characterized by a backbone primarily composed of –OH bonds, rendering it entirely biodegradable.^53^ The realm of biodegradable polymers has witnessed significant advancements, finding applications in diverse fields such as orthopedic surgery, drug delivery, and tissue engineering.^54,55^ Usually, the degradation rate of these materials is carefully controlled by adjusting different physicochemical properties, such as crystallinity, molecular weight, chemical composition, hydrophilicity, and surface area. Polymers that can be degraded in vivo by factors such as body fluids, cellular activities, and enzymatic reactions must yield biocompatible degradation products. Furthermore, these degradation products must be effectively removed from the biological system through resorption. The disintegration time of the material should be synchronized with the healing or regeneration process, and any changes in the mechanical properties of the material resulting from disintegration should not adversely affect the healing or regeneration process.^56^ Certainly, this paradigm is naturally utilized in the processes of tissue generation, remodeling, and regeneration, where cells enzymatically break down the extracellular matrix in their vicinity.^57^ By evaluating and experimenting with a biomaterial responsive to these enzymatic activities, one can strive toward achieving the objective of biomimetic material degradation. To accomplish this, a comprehensive understanding of how specific enzymes impact the degradation kinetics of diverse materials is essential for designing biomaterials with a controlled degradation rate. A schematic representation of the enzymatic and hydrolytic degradation of polymer foam samples is presented in Figure 5.^58^

**Figure 6:**
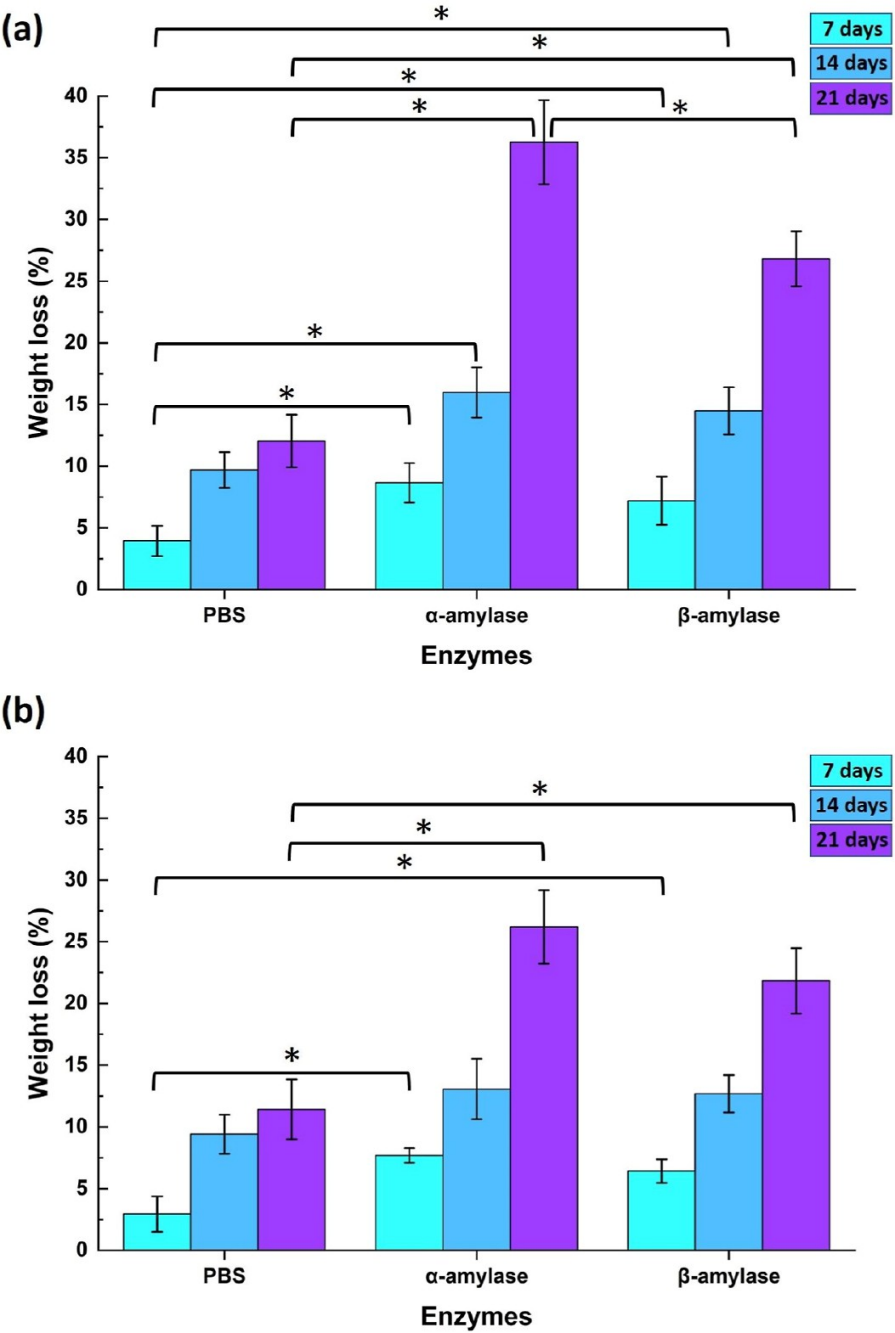
Weight loss (%) for both (a) PVA/starch and (b) PVA/starch/AMX foam samples under different enzymatic/hydrolytic conditions over a span of 3 weeks during the incubation period. The graph provides insight into the dynamic changes experienced by these biomaterials in response to various enzymatic and hydrolytic environments. All data are presented as the mean (standard deviation, ± SD). (*p < 0.05; *statistically significant differences between all pairwise multiple comparison groups).

Upon observation, it was evident that the PVA/starch foam biomaterial exhibited a weight loss of 36.25 ± 3.25% when subjected to α-amylase incubation for 21 days. Similarly, the PVA/starch/AMX foam biomaterial displayed a weight loss of 26.19 ± 2.98% under the same conditions. This suggested substantial degradation of the starch component within the PVA/starch blend. When the β-amylase enzyme was used, a weight loss of approximately 26.80 ± 2.22% was observed for the PVA/starch foam biomaterial, while the PVA/starch/AMX foam biomaterial showed a weight loss of 21.82 ± 2.65% after a 21 day incubation period. These variations in weight loss were attributed to the distinct modes of action exhibited by the two enzymes on the starch molecule, resulting in divergent effects. This data explains the significance of enzyme specificity in influencing the degradation patterns of biomaterials in biological contexts (Figure 6).

Figure 6 also presents the weight loss observed for both samples after 21 days incubated in PBS solution as hydrolytic degradation. The incubation of PVA/starch and PVA/starch/AMX foam samples in all biodegradation solutions leads to an increase in weight loss, with increasing incubation days from 7 to 21 days as expected. A weight loss of 12.4 ± 2.12% was observed in PVA/starch foam samples, and a weight loss of 11.42 ± 2.42% was noted in PVA/starch/AMX foam samples when incubated in a PBS solution over a 21 day period. This phenomenon is attributed to the leaching of plasticizers and other additives employed during processing rather than polymer chain scission.^59^ It is evident that enzymes play a significant role in the degradation of biomaterials, and the incubation with α-amylase results in a greater weight loss compared with that observed with β-amylase, as noted in both foam samples. The primary enzymes responsible for the breakdown of starch include α-amylases, β-amylases, and various debranching enzymes. α-Amylase, specifically, is an endospecific enzyme that facilitates the hydrolysis of α-1,4-glycosidic linkages in starch, producing maltose and dextrins and thereby reducing the molecular size of starch. Additionally, research indicates that α-amylase possesses the capability to degrade chemically modified starch. It is noteworthy that α-amylase is not only present in saliva but can also be found in human blood.^60,61^

When the two synthesized foam materials were compared, PVA/starch displayed higher degradability. This is attributed to the enzyme’s enhanced ability to target the starch component of the blend, a characteristic associated with the physicochemical and morphological structure of the samples. Additionally, the absence of AMX particles in PVA/starch contributes to the higher degradability. Moreover, a higher water absorption rate, particularly in polymeric-based biomaterials, tends to expedite the hydrolysis process. The results presented in this section are further substantiated by the findings in Figure 4.

### In Vitro Drug Loading/Drug Release Profile Study

**Figure 7:**
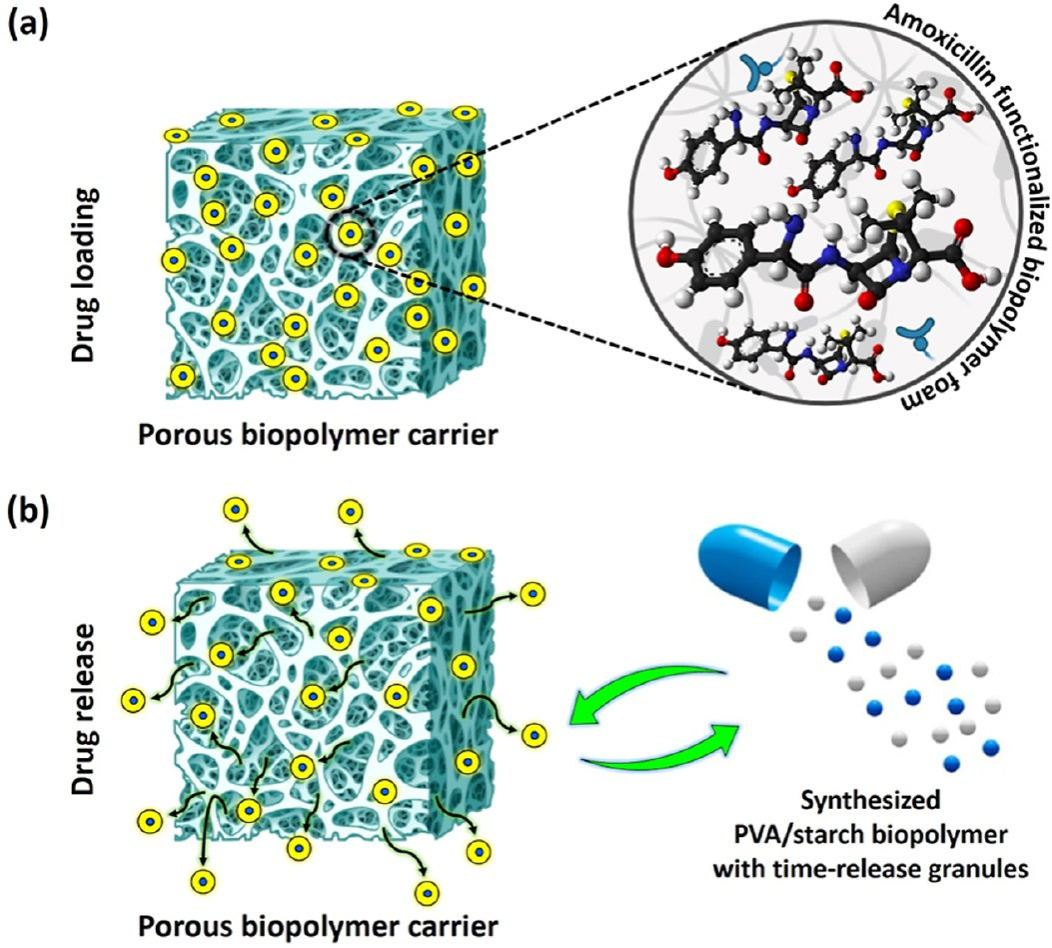
Schematic representations of (a) drug loading in biopolymer foam samples using the vacuum-assisted method and the (b) schematic drawing of the drug release profile aiming to provide controlled drug release.

**Figure 8:**
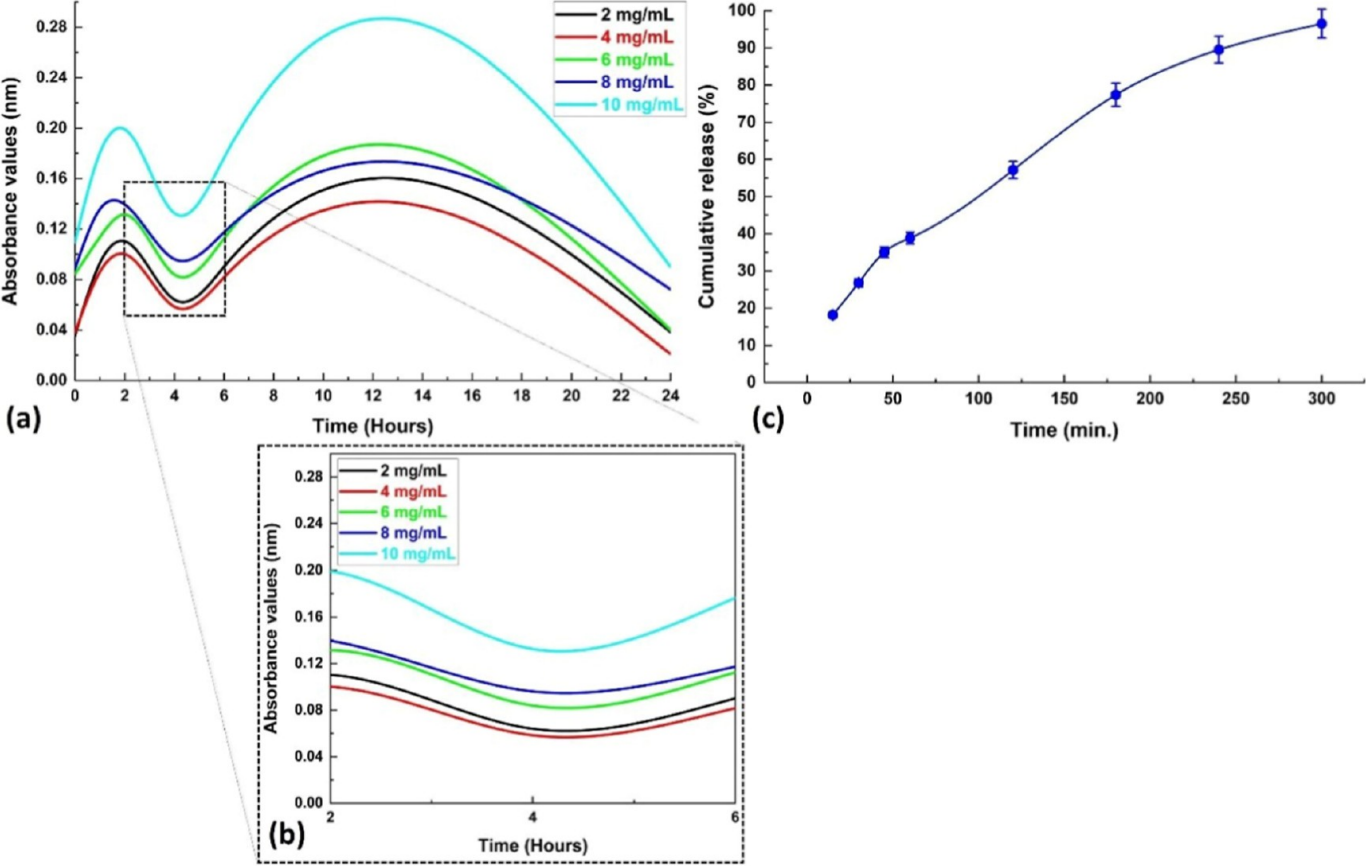
In vitro drug release profiles of PVA/starch/AMX biopolymer foams: (a) absorption spectra of PVA/starch/AMX biopolymer foams at different amounts of AMX drug, (b) detailed inset graph of the complex dynamics of the AMX drug release process over a comprehensive period of between 120 and 360 min, and (c) cumulative release graph of PVA/starch/AMX biopolymer foams. All data are presented as the mean (standard deviation, ± SD).

Drug loading and drug release play pivotal roles in the realm of polymer-foam-based drug delivery systems, serving as critical determinants that influence the efficacy and therapeutic outcomes of the administered pharmaceuticals. Schematic representations of drug loading in polymeric foam samples using the vacuum-assisted method and the drug release profile are provided in Figures 7a,b and 8, respectively. The process of drug loading involves incorporating pharmaceutical agents into the porous structures of polymer foams. This step is crucial for achieving the desired therapeutic effect as it directly impacts the concentration of the drug within the delivery system. Proper drug loading enables precise control of the drug concentration in the polymer matrix. This control is essential for administering accurate and controlled dosages, ensuring optimal therapeutic outcomes while minimizing potential side effects. Effective drug loading sets the stage for controlled-release formulations, allowing for a prolonged and controlled release of the drug over time. This is particularly advantageous for medications requiring a gradual and sustained/controlled therapeutic effect^65,66^ (Figure 7a). Understanding the kinetics of drug release from polymer foams is essential for tailoring release profiles to meet specific therapeutic needs. Different medical conditions may demand rapid, controlled, or delayed release, and the drug release mechanism can be customized accordingly. Controlled drug release helps to enhance patient compliance by reducing the frequency of administrations. This is especially beneficial in chronic conditions, where maintaining a consistent therapeutic concentration is crucial. Precise control over drug release minimizes the risk of adverse effects associated with rapid or excessive drug exposure. This aspect is vital for optimizing the safety profile of the medication (Figure 7b).

Figure 8a,b depicts the absorbance curves of AMX-loaded PVA/starch foams prepared with five different concentrations (2, 4, 6, 8, and 10 mg/mL) at 290 nm. From the onset up until the second hour, there is a marked increase in absorbance values. At the fourth and sixth hours, however, the absorbance values for 2 and 4 mg/mL concentrations decline, while those for higher concentrations persist in increasing. This distinctive pattern strongly suggests that the integration of AMX into the foam sample continues at elevated concentrations but slows down and reaches a saturation point at lower concentrations. Based on the absorbance curves, a drug release study was performed, selecting the 10 mg/mL concentration among the prepared concentrations. Figure 8c presents the intricate dynamics of the AMX drug release process over a comprehensive duration of 300 min, shedding light on the intricate interplay between concentration levels and release kinetics.

### Cumulative Release Behavior of the AMX from the Biofoam Samples

The data presented in Figure 8c illustrate the cumulative release of AMX from the PVA/starch/AMX biofoams over a period of time. The results show a gradual increase in the percentage of AMX released from the biopolymer over time. At the 15 min mark, approximately 18.18 ± 0.72% of the AMX had been released. This percentage increased steadily over time, reaching about 26.81 ± 1.07% at 30 min, 35 ± 1.40% at 45 min, and 38.84 ± 1.54% at 60 min. After 120 min, the release had increased to 57.14 ± 2.28%, and by 180 min, it had reached 77.36 ± 2.09%. At 240 min, the release was at 89.55 ± 3.58%. Finally, by 300 min, approximately 96.53 ± 3.86% of the AMX had been released from the biofoams. These findings indicate that AMX is progressively and steadily released from the biopolymers over time, suggesting a controlled release mechanism. The controlled release can be attributed to the partial degradation of the PVA-based porous biopolymer. The loaded drug distributes within the medium through these pores, and the degradation rate of the polymer layer significantly influences this drug release process. This phenomenon is closely related to the hydrophilic nature of AMX, which readily absorbs water and can penetrate through small pores in the polymer.^34^

### Cytotoxicity of the Biofoam Samples

Cytotoxic properties of biomaterials are a very important property, and cell viability of biomaterials in in vitro environments is usually evaluated by cytotoxicity tests. The cytotoxicity of all biofoam samples was examined after 24 h of incubation with fibroblasts (L929) versus the control group. Figure S4 (Supporting Information) shows a cell viability diagram of biofoam samples from the MTT assay and demonstrates that all groups supported cell proliferation with cell viability values higher than 83%. In the experimental study, it was observed that both materials were not cytotoxic. According to Annex C of ISO 10993-5,^67^ if the relative cell viability for the highest concentration of the test material is ≥ 70%, the material is considered noncytotoxic. The results demonstrated a clear relationship between AMX-loaded PVA/starch biofoam samples and decreased cell viability. For instance, in the PVA/starch group, cell viability was observed at 94.39 ± 1.93%, representing the highest cell activity. Conversely, cell viability slightly decreased to 82.98 ± 2.83% in the PVA/starch/AMX group due to the presence of AMX in the biofoam.^68^

### Antimicrobial Activity of Biofoam Samples

**Figure 9:**
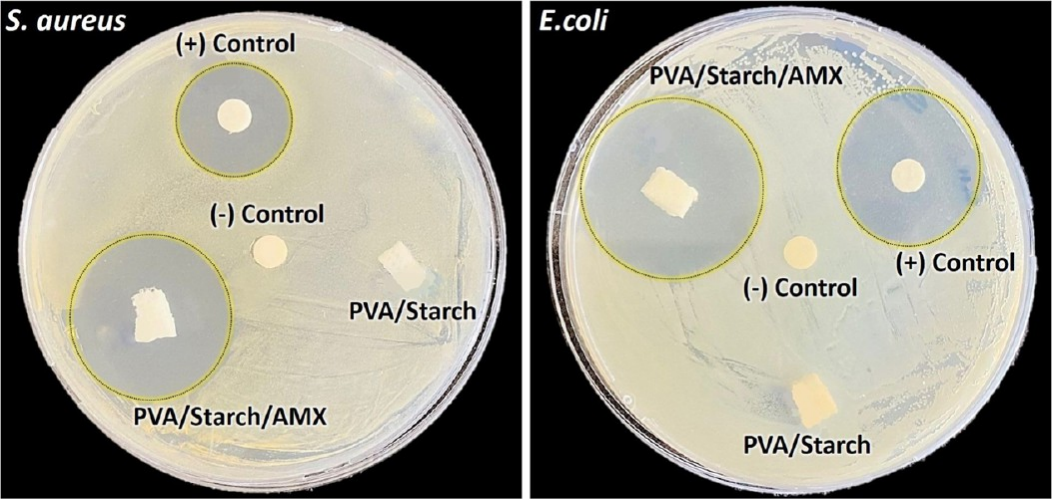
Inhibitory effect of PVA/starch, PVA/starch/AMX biofoam samples, and (+) control and (−) control samples against (a) *S. aureus* and (b) *E. coli* after incubating at 37 °C for 24 h.

The scientific mechanism behind the antimicrobial action of PVA/starch biopolymer foams loaded with AMX involves several factors including the release of the drug, interaction with bacterial cells, and disruption of cellular processes. When AMX is loaded into the PVA/starch biofoams, it can be released gradually over time. As the AMX is released from the biofoams, it comes into contact with bacterial cells. As outlined previously, AMX exhibits bactericidal activity, combating both Gram-positive and Gram-negative microorganisms through the inhibition of bacterial mucopeptide wall biosynthesis and repair.^69^ It has also been observed in literature studies that the combination of biopolymer materials and AMX exhibits a synergistic effect, potentially overcoming the resistance mechanisms displayed by certain bacteria.^70,71^ PVA/starch and AMX-loaded PVA/starch biofoam samples were evaluated for their antimicrobial activity against *S. aureus* and *E. coli* bacteria, as presented in Figure 9. Pure AMX antibiotic (30 μg/disc) served as the positive control (+), while antibiotic discs without AMX were used as negative controls (−). The inhibitory effect against microorganisms was measured using calipers with a sensitivity of 0.01, based on the clear zone surrounding all samples. If no clear zone is observed around the tested samples, then it indicates no inhibitory effect, and the diameter is recorded as zero. Among the successfully synthesized biofoams, PVA/starch/AMX exhibited significant antimicrobial activity against both bacterial microorganisms. The inhibitory zones against *E. coli* and *S. aureus* were measured as 3.57 and 3.06 cm, respectively, for PVA/starch/AMX biofoams. In the (+) control samples, the inhibitory zones against *E. coli* and *S. aureus* were measured as 2.57 and 2.0 cm, respectively (refer to Table 1). When macrophotographs obtained for bacterial growth determination after 24 h of incubation at 37 °C (Figure 9) were examined closely, it was observed that the biofoams prepared without AMX did not exhibit significant antimicrobial activity. Nonetheless, it can be speculated that certain regions of the medium show slight clearing, suggesting that drug-free sponges also possess some level of inhibitory effect on bacterial growth.

**Table 1:** Antimicrobial Activity Data Results of Prepared Samples

samples	*E. coli*	*S. aureus*
PVA/starch/AMX	3.57 cm	3.06
PVA/starch		
positive (+) control	2.70 cm	2.0 cm
negative (−) control		

## Conclusions

In this experimental study, PVA-based biopolymer foams were prepared by using a low-cost and environmentally friendly method by combining different types of natural starch. Using the porous morphologies of the synthesized biofoams, AMX was loaded with the goal of developing a reproducible approach for simultaneous delivery of various antibiotic classes, and all properties were thoroughly investigated. The biofoams exhibited a fibril structure with spindle-like and partially spherical pores, along with lump-shaped starch granules dispersed throughout. The incorporation of AMX in PVA/starch/AMX biofoams resulted in a rougher surface texture and an open-cell configuration, showcasing potential applications in drug delivery systems emphasizing effective drug entrapment and controlled release. The swelling behavior of the PVA/starch foams exhibited accelerated swelling rates and increased equilibrium swelling ratios compared to the PVA/starch/AMX biofoams, attributed to chain disentanglement and disruption of hydrogen bonding between polymer molecules during extended immersion times. The enzymatic and hydrolytic degradation studies revealed that PVA/starch biofoams exhibited higher weight loss percentages compared to PVA/starch/AMX biofoams when subjected to α-amylase and β-amylase incubations, highlighting the significant impact of enzyme specificity on biomaterial degradation. The drug release study revealed a gradual and consistent release of AMX from PVA/starch/AMX biofoams over time, with approximately 96.53% of the drug released by 300 min, demonstrating a controlled release mechanism influenced by the gradual degradation of the porous biopolymer and the physical and chemical structure of AMX. This controlled release is indicative of the interplay between drug distribution within the medium and the degradation rate of the polymer layer. The MTT assay showed that all groups supported cell proliferation, with cell viability above 83%, indicating noncytotoxicity. The PVA/starch/AMX biofoams exhibited significant antimicrobial activity against both Gram-positive (*S. aureus*) and Gram-negative (*E. coli*) bacteria, with inhibitory zones measuring 3.57 cm for *E. coli* and 3.06 cm for *S. aureus*. This activity was attributed to the gradual release of AMX from the biopolymer, potentially overcoming the bacterial resistance mechanisms. In contrast, biofoams without AMX showed minimal antimicrobial activity, suggesting inherent inhibitory effects even in drug-free samples. This sustainably synthesized PVA/starch/AMX biofoam material demonstrates promising potential as a drug carrier system for biomedical applications, characterized by its controlled drug release, significant antimicrobial activity, and environmentally friendly synthesis method.

## Supporting Information Available

Detailed methods and key experimental data that support the original findings (PDF)
