# Supplementary material for: Sustainable Synthesis of Multifunctionalized Amoxicillin-Loaded Biopolymer Foams
Source: ACS Omega. 2025 Apr 11;10(15):15525–39. doi: 10.1021/acsomega.5c00442 (PMC12019502; doi:10.1021/acsomega.5c00442)
Supplement: Supplementary file 1 — ao5c00442_si_001.pdf [file ao5c00442_si_001.pdf]

Supporting Information

Sustainable Synthesis of Multifunctionalized Amoxicillin-Loaded Biopolymer Foams

*Kerim Emre Öksüz[1,2], Saynur Arslan[1]

[1]Department of Metallurgical and Materials Engineering, Sivas Cumhuriyet University, Sivas, 58140, Türkiye

[2]Institute of Science and Technology, Department of Bioengineering, Sivas Cumhuriyet University, Sivas, 58140, Türkiye

*To whom correspondence should be addressed. Tel:+90 346 487 00 00 / Room number: 2806

Email: *kerimemreoksuz@gmail.com* - *emre.oksuz@cumhuriyet.edu.tr*

Sustainable synthesis of polymeric biofoam samples

A detailed schematic representation of the possible hydrogen bond formations resulting from molecular interactions between starch and PVA polymer, as well as the step-by-step synthesis of porous biofoams, is provided below in Figure S1 and Figure S2, respectively.

Supplementary Figures

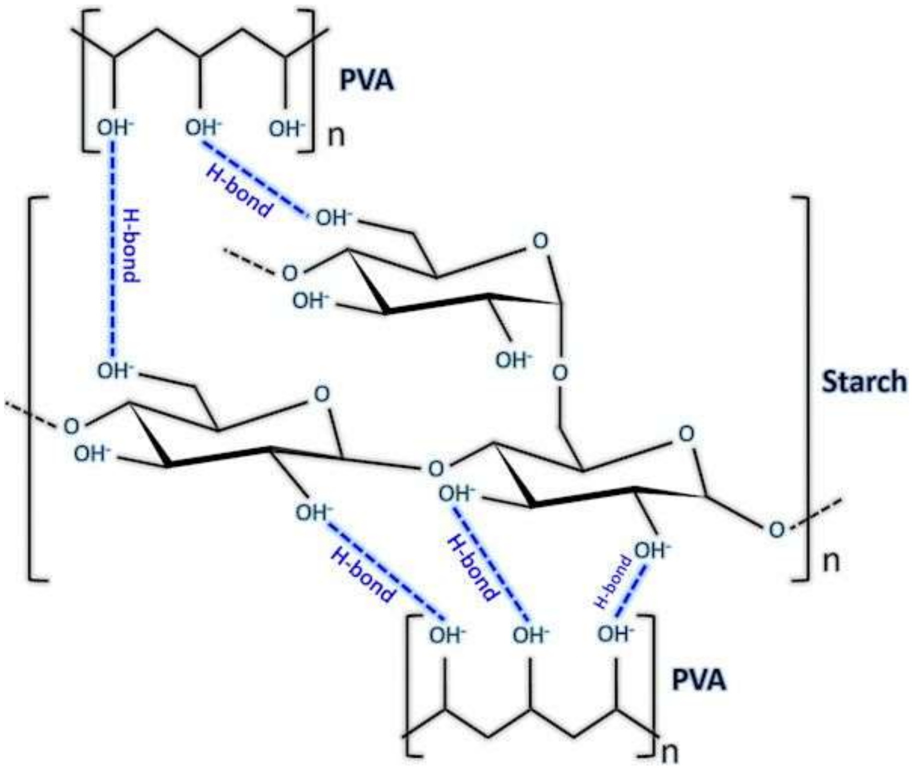

Figure S1. The possible formation of hydrogen bonds between starch and PVA. The rationale behind this bonding lies in the shared characteristic of both starch and PVA as polar substances, each featuring hydroxyl groups (-OH) within its chemical structure. Given the inherently polar nature of these hydroxyl groups, there is a propensity for the formation of both intermolecular and intramolecular hydrogen bonds. This bonding phenomenon provides to enhance the structural cohesion and integrity of PVA-starch blends. The interplay of these hydrogen bonds contributes to the overall stability and properties of the blended material, showcasing the significance of molecular interactions in the context of PVA-starch compatibility [1,2]

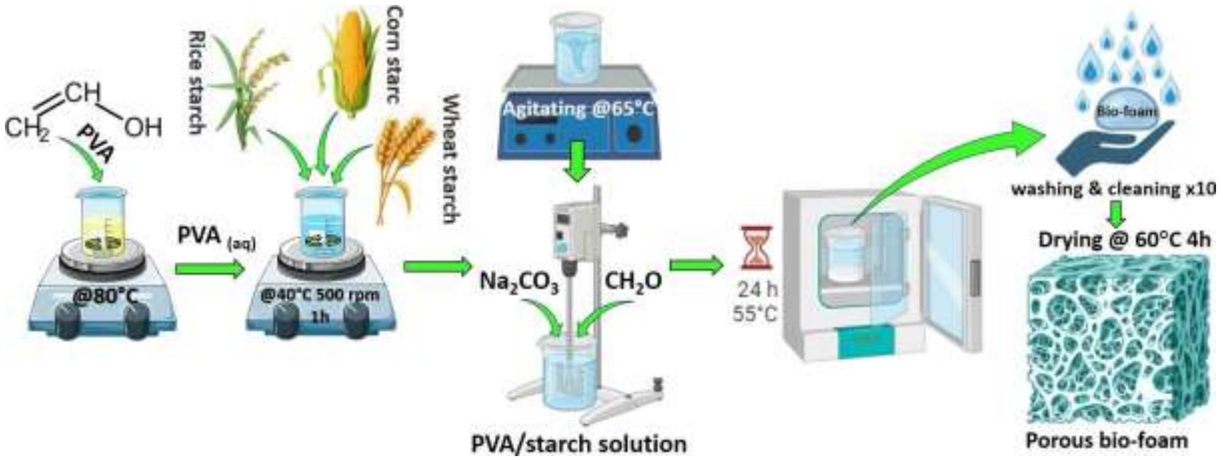

Figure S2. The arrangement of the sustainable synthesis process for the PVA/starch biofoam encompasses various stages. This synthesis procedure encompasses a sequence of steps, including mixing, stirring, foaming, stabilizing, and drying, all meticulously executed to achieve a uniform and homogeneous mixture. The coordination of these processes is essential in ensuring the successful development of the biofoam with optimal properties

Results and Discussion

EDXS Analyses

The surface composition of the compounds/particles on the PVA/starch and PVA/starch/AMX bio-foam samples was analyzed using FE-SEM-EDXS spectra. These spectra and their corresponding calculated values are shown in Figure S3.

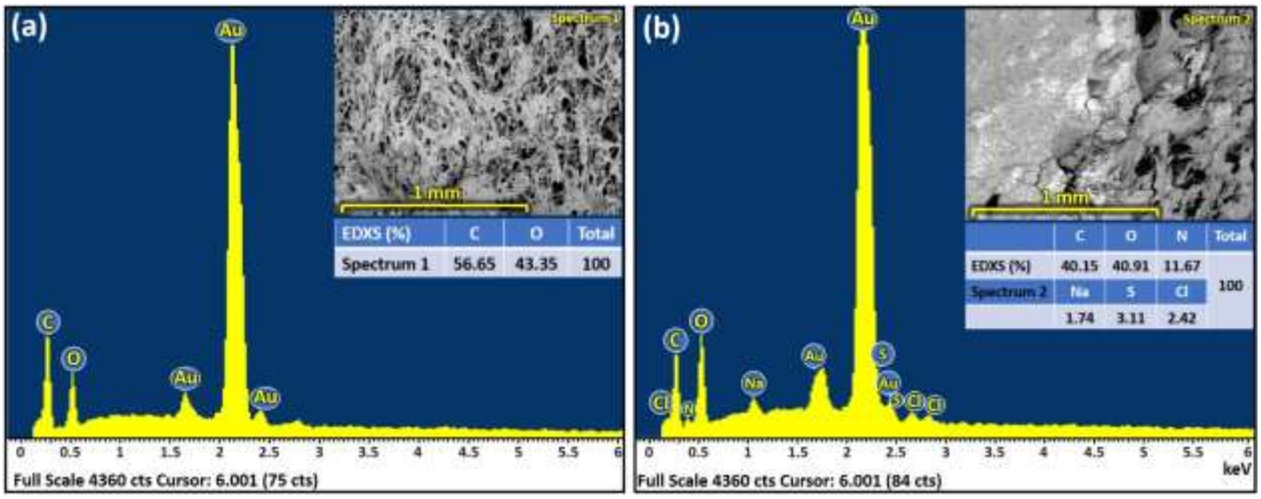

Figure S3. EDXS spectra obtained from the FE-SEM images of the surfaces of both (a) PVA/starch and (b) PVA/starch/AMX biofoam samples. These spectra provide information about the elements present on the biofoam surface and their distribution

Cytotoxicity of the biofoam samples

The cell viability values for each group, obtained after incubating synthesized biofoams with L929 mouse cell lines for 24 hours, are presented in Figure S4. The statistical analysis results of the differences between these values and those of the control group, for samples maintained under identical environmental conditions, are also provided in the same graph.

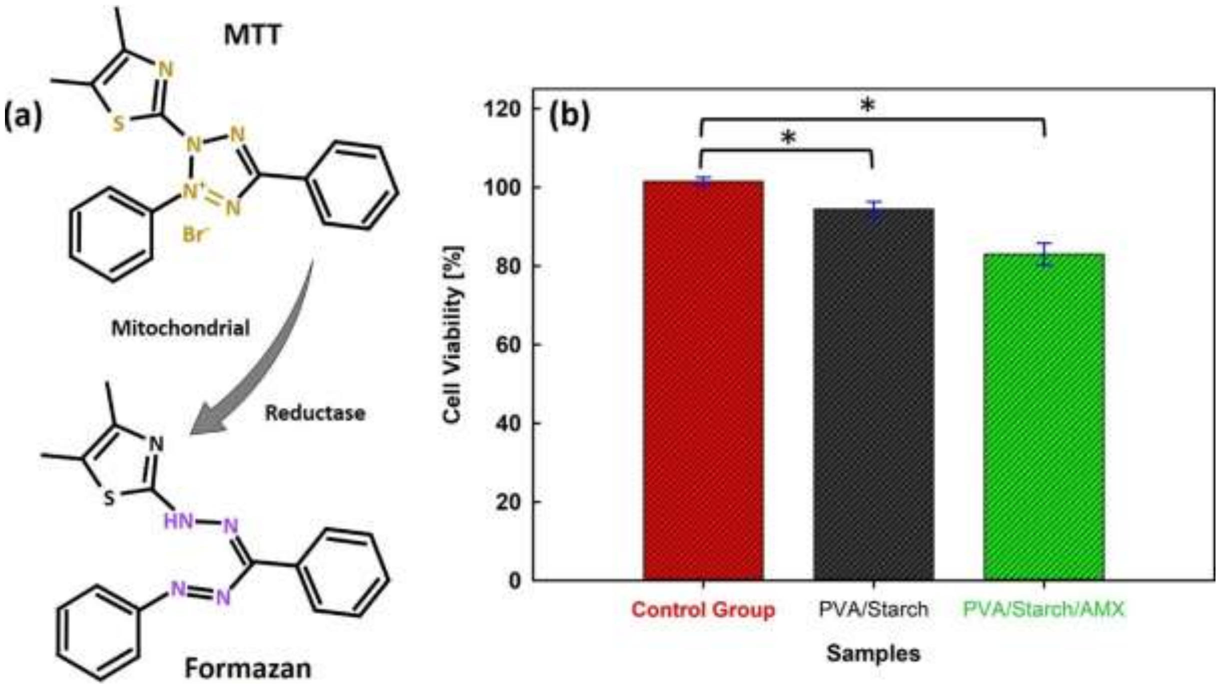

Figure S4. (a) Colorimetric reaction between 3-(4,5-dimethyl-2-thiazolyl)-2,5-diphenyl-2H-tetrazolium bromide and (E,Z)-5-(4,5-dimethylthiazol-2-yl)-1,3-diphenyl-formazan in MTT assay, (b) Cell viability (%) results as determined by the MTT assay. Values are expressed as the mean ± SD from three (*n=3*) independent experiments (*$p < 0.05$; *indicates statistically significant differences between groups)

References

[1] Yu, X., Lin, L., Mei, L., Sun, C., Zhu, Z., Du, X., & Chen, P. (2021). Development, characterization, and antioxidant evaluation of corn starch- based composite films containing tea polyphenols. *Journal of Applied Polymer Science*, *139* (15). https://doi.org/10.1002/app.51928

[2] Musa, B. H., & Hameed, N. (2021). Effect of crosslinking agent (glutaraldehyde) on the mechanical properties of (PVA/Starch) blend and (PVA/PEG) binary blend films. *Journal of Physics Conference Series*, *1795*(1), 012064. https://doi.org/10.1088/1742-6596/1795/1/012064
